# Overview of the English Longitudinal Study of Ageing

**DOI:** 10.1093/geroni/igaa057.2202

**Published:** 2020-12-16

**Authors:** Andrew Steptoe

**Affiliations:** University College London, London, England, United Kingdom

## Abstract

The English Longitudinal Study of Ageing (ELSA) is a unique and rich resource of information on the health, social, wellbeing and economic circumstances of the English population aged 50 and older. The current sample contains data from up to nine waves of data collection covering a period of seventeen years (2002-2019). The multidisciplinary and longitudinal nature of the data allows the examination of complex relationships and causal processes. The survey data are designed to investigate a broad set of topics to help understand the ageing process. These include predictors of well-being, health trajectories, disability and healthy life expectancy, the determinants of economic position in older age, the links between socioeconomic status, physical health and functioning, cognition and mental health, the nature and timing of retirement and post-retirement labor market activity, household and family structure, social networks and social supports; patterns, determinants and consequences of social, civic and cultural participation.

